# Depletion of PCAT-1 in head and neck cancer cells inhibits tumor growth and induces apoptosis by modulating c-Myc-AKT1-p38 MAPK signalling pathways

**DOI:** 10.1186/s12885-019-5562-z

**Published:** 2019-04-15

**Authors:** Subhayan Sur, Hiroshi Nakanishi, Robert Steele, Ratna B. Ray

**Affiliations:** Department of Pathology, Saint Louis University, 1100 South Grand Boulevard, St. Louis, MO 63104 USA

**Keywords:** Head and neck squamous cell carcinoma (HNSCC), Long non-coding RNA, PCAT-1, c-Myc-AKT1signaling, Tumorigenicity

## Abstract

**Background:**

Head and neck squamous cell carcinoma (HNSCC) represents one of the most common malignancies worldwide with a high mortality rate mainly due to lack of early detection markers, frequent association with metastasis and aggressive phenotype. Recently, long non-coding RNAs (lncRNAs) have been shown to have important regulatory roles in human cancers. The lncRNA prostate cancer-associated transcript 1 (PCAT-1) showed potential oncogenic roles in different cancers, however its role in HNSCC is not known. In this study, we evaluated the role of the PCAT-1 in HNSCC.

**Methods:**

The expression of PCAT-1 was measured by quantitative real-time PCR in 23 paired human HNSCC tissues and adjacent non-tumor tissue specimens. Cell proliferation after depleting PCAT-1 was determined. Effect of PCAT-1 depletion in HNSCC cell lines was determined by qRT-PCR and Western blot analyses. Finally, JHU029 HNSCC cells was implanted subcutaneously into athymic nude mice and therapeutic potential of PCAT-1 was investigated.

**Results:**

Up-regulation of PCAT-1 in TCGA dataset of HNSCC was noted. We also observed increased expression of PCAT-1 in archived HNSCC patient samples as compared to adjacent non-tumor tissues. Knockdown of PCAT-1 significantly reduced cell proliferation in HNSCC cell lines. Mechanistic study revealed significant down regulation of c-Myc and AKT1 gene in both RNA and protein levels upon knockdown of PCAT-1. We observed that c-Myc and AKT1 positively correlate with PCAT-1 expression in HNSCC. Further, we observed activation of p38 MAPK and apoptosis signal-regulating kinase 1 upon knockdown of PCAT-1 which induces Caspase 9 and PARP mediated apoptosis. Targeted inhibition of PCAT-1 regresses tumor growth in nude mice.

**Conclusion:**

Together our data demonstrated an important role of the PCAT-1 in HNSCC and might serve as a target for HNSCC therapy.

## Background

Head and neck squamous cell carcinoma (HNSCC) represents one of the most common malignancies worldwide with a high mortality rate mainly due to lack of early detection markers, frequent association with metastasis and aggressive phenotype [1]. The standard treatment for HNSCC is platinum-based drugs, 5-FU, and cetuximab regimen [2, 3]. Other agents like isotretionin, celecoxib, and erlotinib were in randomized clinical trials; however, no effective and tolerable agent has been identified for prevention of HNSCC [2]. Thus, there is an urgent need to identify biomarkers and therapeutic targets for this disease.

The recent discovery of long non-coding RNAs (lncRNAs) has gained widespread attention as a new layer of regulation in biological processes. It has been shown that lncRNAs, over 200 nucleotides in size, regulate gene functions either interacting with DNA, RNA or directly with proteins [1, 4]. Dysregulation of lncRNAs were found to be associated with various human diseases ranging from neurodegeneration to cancer [1]. To date, numerous cancer-associated lncRNAs are reported to modulate tumour growth, invasion and metastasis, and have been implicated as potential alternative biomarkers and therapeutic targets for cancer [1]. Prostate cancer-associated transcript 1 (PCAT-1) was initially identified in prostate cancer [5, 6]. PCAT-1 is located at the Chr8q24 gene desert approximately 725 kb upstream of the Myc oncogene, the region is frequently amplified in HNSCC [7–9]. Overexpression of PCAT-1 was reported in several cancers and its function is associated with cell proliferation, invasion, metastasis, survival, cell cycle, chemoresistance, and homologous recombination. [7]. However, the role of PCAT-1 in HNSCC remains unknown.

In this study, we observed overexpression of PCAT-1 in HNSCC patient samples. Knockdown of PCAT-1 inhibited HNSCC cell proliferation. We observed that depletion of PCAT-1 in HNSCC cell lines inhibits c-Myc and AKT1 genes and activates p38 MAPK signalling, resulting in Caspase 9 mediated apoptosis. Targeted inhibition of PCAT-1 reduced in-vivo tumorigenesis in mouse xenograft model. To our knowledge this is first study demonstrating an important role of PCAT-1 in HNSCC.

## Methods

### Patient tissue specimens

Achieved frozen tissues of HNSCC and matched adjacent non-tumor tissues (*n* = 23) were obtained from Saint Louis University Cancer Center. All the samples were immediately snap frozen in liquid nitrogen for long-term preservation until RNA extraction.

### Cell culture and siRNA transfection

HNSCC cell line Cal27 was purchased from the ATCC. JHU029 and JHU022 cell line were obtained from the Johns Hopkins University through MTA. Cal27 cells were maintained in DMEM media, JHU029 and JHU022 cells were in RPMI- 1640 media, and supplemented with 10% FBS and 1% penicillin/ streptomycin in a humidified CO_2_ incubator at 37 °C. Normal oral keratinocytes (NOK) cells (kindly gifted by Karl Mugner) were maintained in Keratinocyte SFM medium supplemented with epidermal growth factor and bovine pituitary extract (GIBCO, Life technologies). All cells are mycoplasma free, but have not authenticated further.

JHU029 and Cal27 cells (2.5 × 10^5^) were plated using 6-well plates overnight in antibiotic free media. Cells were transfected with either control oligo or siRNAs to PCAT-1 (Assay ID: n511583, ThermoFisher Scientific) mixed with Opti-MEM and LipofectamineRNAiMAX (Invitrogen) according to manufacturer’s protocols for a final concentration of siRNA of 50 nM, and incubated for 48 h. All the analysis was performed in triplicate.

### Cell proliferation assay

JHU029 and Cal27 cells were plated using 35 mm dishes and transfected with/ without siRNA. At 0 h, 48 h and 96 h after siRNA transfection cells were harvested with trypsin, stained with trypan blue, and percentage of live cells were counted using a haemocytometer. The cell numbers in control or transfected were compared with the number at 0 h. The experiment was performed in triplicate.

### RNA isolation and expression analysis

Total RNA was extracted by TRIzol reagent followed by cDNA synthesis using 1 μg of total RNA by SuperScript III Reverse Transcriptase (Life technology, USA) in accordance with the manufacturer’s protocol. Real-time PCR was performed for quantitation of gene expression using TaqMan Universal PCR master mix and 6-carboxyfluorescein (FAM)-MGB probes for PCAT-1 (assay ID:Hs04275836_s1; ThermoFisher Scientific) as per manufacture’s protocol. 18 s rRNA (assay ID:Hs03928985_g1; ThermoFisher Scientific) was used as an endogenous control. Gene expression analysis of c-Myc (FR: 5′ GCTCGTCTCAGAGAAGCTGG 3′ and RP: 5’GCTCAGATCCTGCAGGTACAA 3′) and AKT1 (FR: 5’TCTATGGCGCTGAGATTGTG 3′ and RP: 5’CTTAATGTGCCCGTCCTTGT 3′) was carried out by SYBR green based detection system as per standard procedure. GAPDH (FR: 5′ CATGTTCGTCATGGGTGTGAACCA 3′ and RP: 5′ AGTGATGGCATGGACTGTGGTCAT 3′) or 18 s (FP: 5′ GTCATAAGCTTGCGTTGATT 3′ and RP: 5′ TAGTCAAGTTCGACCGTCTT 3′) was used as endogenous control. The relative gene expression was analysed by 2^-∆∆CT^ method. Each sample was loaded in triplicate.

### Western blot analysis

Cell lysates were prepared using 2× SDS sample buffer, and western blot analysis was performed using specific antibody to c-Myc (1:1000), AKT1 (1:1000), phospho-P38 (Thr180/ Tyr 182) (1:1000), total P38 (1:1000), phospho- ASK1 (Ser83) (1: 1000), total ASK1 (1: 1000), Caspase-9 (1:1000), and PARP (1:1000). All the primary and HRP-conjugated anti mouse or anti-rabbit secondary antibodies (1:2000) were purchased from Cell Signalling Technology. The blot was reprobed with either Actin or GAPDH antibody (1:5000) to compare protein load in each lane. Densitometry analysis was done using Image J software.

### Xenograft tumor growth

JHU029 cells were trypsinized, washed, and resuspended in PBS containing 40% BD-Matrigel. Cells (1.4X10^6^) were injected subcutaneously into the flank of BALB/c athymic nude female mice (6 weeks old, *n* = 6). Mouse number are chosen based on our previous experience. After three weeks of palpable tumor developed (>75mm^3^), 10 μg of siPCAT-1 or control oligo complexed with siPORTamine (Invitrogen) in 50 μl Opti-MEM were injected intratumorally at an interval of 4 days a total of ten times. Tumors were measured using a Slide Calliper once a week and volume was calculated using the formula V = ½ (L x W^2^). All the mice were sacrificed at 8-week post injection. After sacrifice tumors were dissected out and snap frozen in liquid nitrogen for RNA extraction and protein isolation. No adverse effect was observed in the animals. Mice were euthanized by asphyxiation by CO2 inhalation using a compressed gas source. This method of euthanasia is consistent with the recommendations of the American Veterinary Medical Association (AVMA) Guidelines for the Euthanasia of Animals. All animal experiments were carried out in accordance with NIH guidelines, following a protocol approved by the Institutional Animal Care and Use Committee (IACUC) of Saint Louis University.

### Bioinformatics and statistical analysis

PCAT-1 gene alteration and expression was analysed from TCGA data sets using cBioPortal (www.cbioportal.org) platform and GEPIA (http://gepia.cancer-pku.cn). *P* value of < 0.05 was considered as cut off value for gene expression analysis. The relationship between PCAT-1 and c-Myc; PCAT-1 and AKT1 in patient samples were evaluated by Pearson’s correlation coefficient (R) (linear regression) using GraphPad Prism 6. Correlation analysis was also performed from the TCGA data set using GEPIA. Results are presented as means ± standard deviations. Data were analysed by Student’s t test. *P* value of < 0.05 was considered as statistically significant.

## Results

### Overexpression of PCAT-1 in HNSCC

**Fig. 1:**
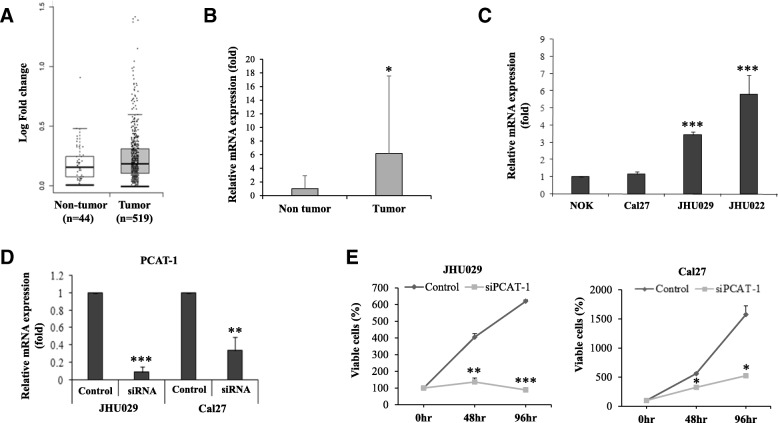
PCAT-1 expression is enhanced in HNSCC and regulates cell proliferation. **a**: PCAT-1 expression in HNSCC patient samples compared to the non-tumor tissues from the TCGA data. *P* value < 0.05 was selected as cut off. **b**: Relative mRNA expression of PCAT-1 gene in HNSCC patient samples (*n* = 23) compared to adjacent non-tumor tissues. 18 s gene used as internal control. **c**: Relative mRNA expression of PCAT-1 in HNSCC cell lines compared to NOK. 18 s gene used as internal control. **d**: JHU029 and Cal27 cells were transfected with PCAT-1 siRNA (50 nM) and after 48 h, PCAT-1 knockdown efficiency was checked by analysing mRNA expression. 18 s gene was used as internal control. **e:** JHU029 and Cal27 cells were transfected with/ without siRNA. At 0 h, 48 h and 96 h cell were stained with trypan blue and percentage of live cells was counted using a haemocytometer. Small bar indicates standard error (*, *p* < 0.05; ** *p* < 0.01; ***p < 0.001)

To understand the clinical relevance of PCAT-1 in HNSCC, we first analysed PCAT-1 expression from TCGA data set. Overexpression of PCAT-1 was seen in tumor samples (*n* = 519) compared to non-tumor tissues (*n* = 44) (Fig. 1a). Out of a total of 783 samples in the TCGA data set 12% of samples showed PCAT-1 gene amplification (cBioPortal). Kaplan–Meier analysis showed survival of patients having PCAT-1 gene alteration (61 total cases) is 32.19 months whereas cases without alteration (440 total cases) in the genes is 56.9 months (cBioPortal). This indicates PCAT-1 alteration might be associated with poor overall survival. We examined PCAT-1 expression in archived HNSCC patient samples and observed a significant increase in PCAT-1 expression in HNSCC tumor samples (*n* = 23) compared to the adjacent non-tumor tissue (Fig. 1b). However, we could not stratify the data with the stage of the tumors, since our sample size was small. All the samples were from tongue origin. We further examined PCAT-1 expression in a panel of human HNSCC cell lines and detected higher expression of PCAT-1 in JHU029 and JHU022 cells when compared with NOK cells (Fig. 1c). This observation suggested that PCAT-1 may act as an oncogene in HNSCC.

### Knockdown of PCAT-1 inhibits HNSCC cell growth and reduces c-Myc and AKT1 expression

**Fig. 2:**
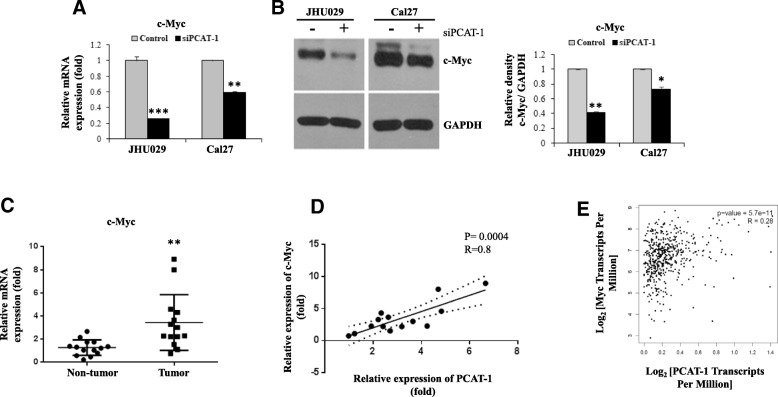
Targeting PCAT-1 inhibits c-Myc. **a**: Relative mRNA expression of c-Myc analysed by qRT-PCR in JHU029 and Cal27 cells with or without transfection of siRNA to PCAT1. Control siRNA was used in parallel. GAPDH gene was used as internal control. **b**: Cell lysates from JHU029 and Cal27 cells with control or siRNA to PCAT-1 were subjected to Western blot analysis for the c-Myc using specific antibody. The membrane was reprobed with antibody to GAPDH as an internal control. Right panel is quantitative representation of Western blot band intensities using Image-J software. **c**: Relative mRNA expression of c-Myc in HNSCC tumors (*n* = 14) compared to the adjacent non-tumor tissues analysed by qRT-PCR. 18 s gene used as internal control. **d**: Correlation analysis of PCAT-1 and c-Myc expression in HNSCC tumor samples (*n* = 14). The relationship was evaluated by Pearson’s correlation coefficient (linear regression) with 95% confidence. **e**: *In-silico* correlation analysis of expression between PCAT-1 and c-Myc in the HNSCC patient samples of TCGA data analysed by GEPIA (http://gepia.cancer-pku.cn). Small bar indicates standard error (*, *p* < 0.05; ** *p* < 0.01; ***p < 0.001)

We depleted the PCAT-1 in JHU029 and Cal27 cells using specific siRNA. As expected, PCAT-1 RNA was decreased significantly in both the cell lines as compared to the control (Fig. 1d). We initially used several siRNAs to PCAT-1, however only one (used in this study) displayed significant reduction of PCAT-1. We provided the assay ID information in the method section. Knockdown of PCAT-1 significantly reduces cell growth in both JHU029 and Cal27 cell lines compared to their respective controls (Fig. 1e). PCAT-1 regulates c-Myc expression in prostate cancer cell lines [5]. We next examined the status of c-Myc in HNSCC cell lines, and observed a significant downregulation of c-Myc at RNA and protein levels in PCAT-1 depleted JHU029 and Cal27 cell lines (Fig. 2a & b), in agreement with earlier observation [7]. We examined c-Myc mRNA expression in HNSCC patient samples (*n* = 14), and found a significant overexpression in tumors compared to adjacent non- tumor tissue (Fig. 2c). The c-Myc expression was positively and significantly correlated with PCAT-1 expression in the patient samples (Fig. 2d). Similarly, in the TCGA dataset, we found a significant positive correlation in expression between PCAT-1 and c-Myc in HNSCC (Fig. 2e).

**Fig. 3:**
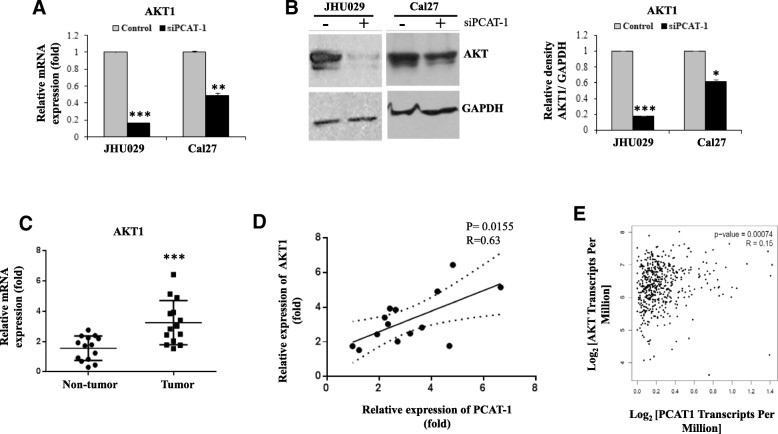
Targeting PCAT-1 inhibits AKT1. **a**: Relative mRNA expression of AKT-1 analysed by qRT-PCR in JHU029 and Cal27 cells with or without transfected with siRNA. Control siRNA was used in parallel. GAPDH gene was used as internal control. **b**: Cell lysates from JHU029 and Cal27 cells transfected with control or siRNA of PCAT-1 were subjected to Western blot analysis for the AKT1 using specific antibody. The membrane was reprobed with antibody to GAPDH as an internal control. Right panel shows quantitative representation of Western blot band intensities using Image-J software. **c**: Relative mRNA expression of AKT1 in HNSCC tumors (*n* = 14) compared to the adjacent non-tumor tissues analysed by qRT-PCR. 18 s gene used as internal control. **d**: Correlation analysis of PCAT-1 and AKT1 expression in HNSCC tumor samples (*n* = 14). The relationship was evaluated by Pearson’s correlation coefficient (linear regression) with 95% confidence. **e:** *In-silico* correlation analysis of expression between PCAT-1 and AKT in the HNSCC patient samples of the TCGA dataset analysed by GEPIA (http://gepia.cancer-pku.cn). Small bar indicates standard error (*, *p* < 0.05; ** *p* < 0.01; ***p < 0.001)

Transcriptome array in PCAT-1 knockdown prostate cancer cells vs. control parental cells identified several PCAT-1 regulated genes [6]. We chose to examine the proliferation associated genes AKT1 and STAT1, since we observed less proliferation in PCAT-1 depleted HNSCC cells. Downregulation of AKT1 mRNA and protein expression was observed in both PCAT-1 depleted HNSCC cell lines (Fig. 3a & b). A significantly higher expression of AKT1 mRNA was also observed in HNSCC patient samples (*n* = 14), and a significant positive correlation was noted between AKT1 and PCAT-1 (Fig. 3 c & d). Similarly, in the TCGA dataset, we found a significant positive correlation in expression between PCAT-1 and AKT1 in HNSCC (Fig. 3e). However, we did not observe alteration of STAT1 expression in HNSCC cell lines upon PCAT-1 knockdown (data not shown). Our result indicates that PCAT-1 regulates c-Myc and AKT1 expression in regulation of HNSCC cell growth.

### Knockdown of PCAT-1 activates p38 MAP kinase signalling and induces apoptosis

**Fig. 4:**
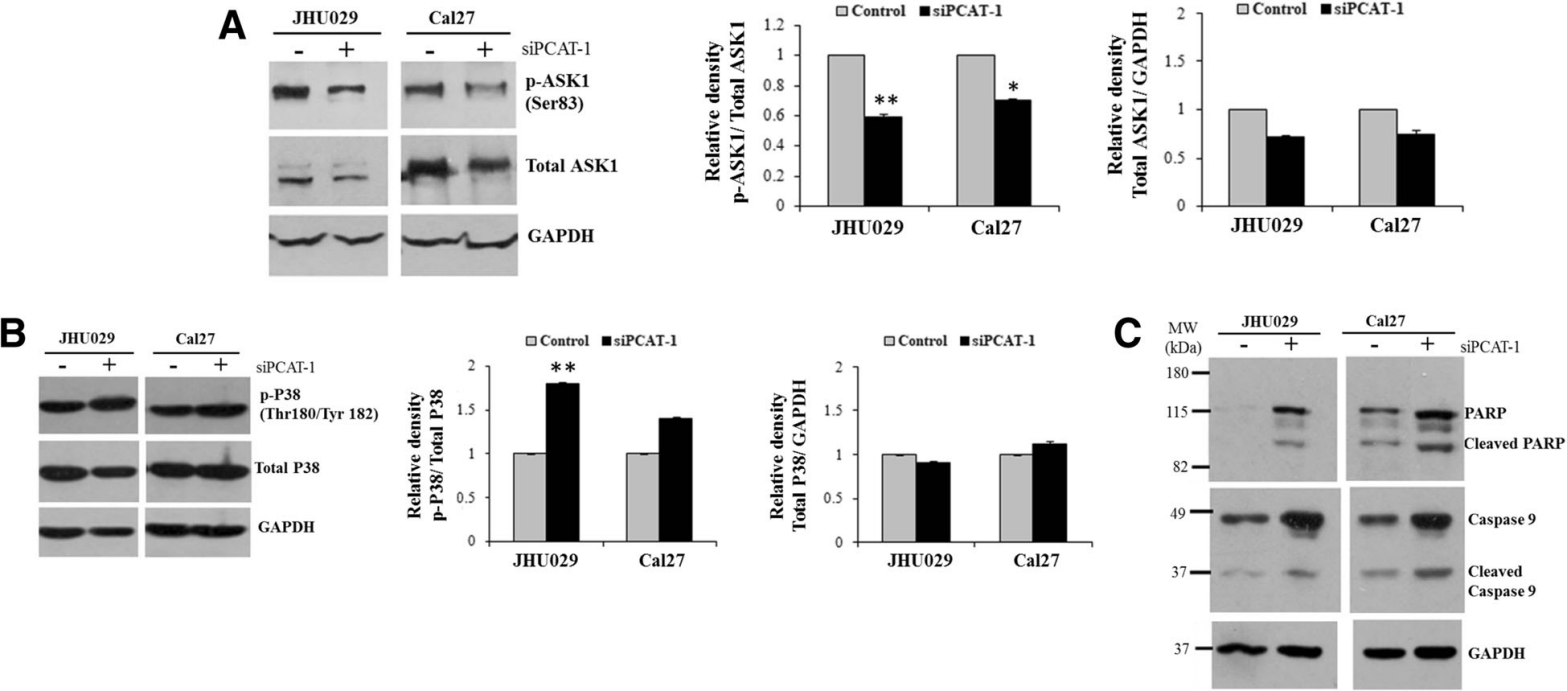
Depletion of PCAT-1 activates p38 MAPK through ASK1 and induces Caspase mediated apoptosis in HNSCC. Protein lysates from JHU029 and Cal27 cells transfected with control or siRNA to PCAT-1 were subjected to Western blot analysis using specific antibodies. **a:** Expression of phospho-ASK1 and total ASK1 and the right panel shows quantitation of the data using Image J Software **b:** Expression of phospho-p38 and total p38 and right panel shows quantitation of the data. The membranes were reprobed with antibody to GAPDH as an internal control. Small bar indicates standard error (*, *p* < 0.05; ** *p* < 0.01). **c:** Western blot analysis of the cell lysates was performed using the specific antibodies to PARP or Caspase 9. The membrane was reprobed with antibody to GAPDH as an internal control

Blockade of AKT signalling induces p38 MAP kinase activation through phosphorylation at Thr 180/Tyr 182 sites by apoptosis signal-regulating kinase 1 (ASK1), a mitogen-activated protein kinase kinase kinase [10–14]. AKT1 inactivates ASK1 by phosphorylating at Ser83 residue. Studies also indicated that activated p38 induces Caspase 9 to induce mitochondria mediated cellular apoptosis [15, 16]. AKT inhibition also directly induces mitochondria mediated apoptosis by activating Caspase 9 [17, 18]. We hypothesized that AKT inhibition upon knockdown of PCAT-1 activates the ASK1- p38 MAPK- Caspase signalling cascade resulting apoptosis in HNSCC. For this, we depleted PCAT-1 by siRNA in JHU029 and Cal27 cells and we observed downregulation of the inactive form of ASK1 (phospho- ASK1- ser83) in both the cell lines (Fig. 4a). Increased expression of phospho p38 MAPK was seen in PCAT-1 depleted HNSCC cells compared to control cells (Fig. 4b). Significant change was seen in JHU029 cells only. This indicates that AKT1 inhibition by PCAT-1 knockdown might induce p38 MAPK activation through ASK1 in HNSCC. Next we checked Caspase 9 and PARP expression in the cell lines. We observed increased expression of cleaved Caspase 9 and PARP upon knockdown of PCAT-1 in both the cell lines, indicating induction of apoptosis (Fig. 4c). These data suggested that inhibition of PCAT-1 induces apoptosis by regulating AKT1- p38 MAPK signalling in HNSCC cell lines.

### Therapeutic potential of PCAT-1 in HNSCC xenograft model

**Fig. 5:**
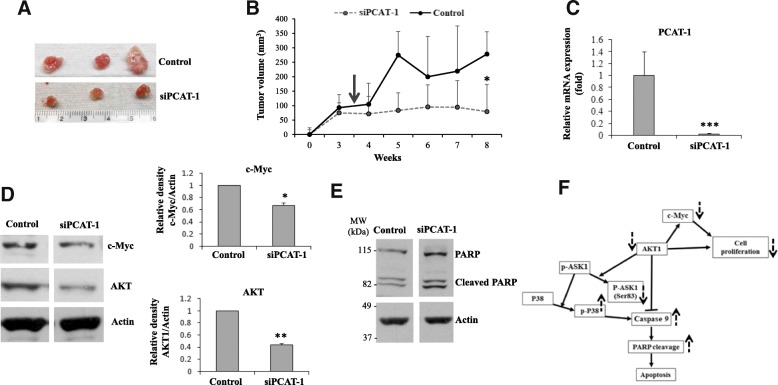
Therapeutic potential of PCAT-1 in HNSCC xenograft model. **a**: JHU029 cells (1.4 × 10^6^) cells were implanted subcutaneously into the flank of nude mice. After three weeks of palpable tumors (~ 75–90 mm^3^), 10 μg of siRNA to PCAT-1 or control oligoes were injected intratumorally at an interval of 4 days for a total of ten times. Representative images of tumors in different groups. **b**: Tumors were measured using a slide calliper, and tumor volumes were calculated (first delivery is indicated by arrow). Small bar indicates standard error (*, *p* < 0.05). **c:** Expression of PCAT-1 was examined from RNA of experimental and control tumors by qRT-PCR. 18 s gene was used as internal control. Small bar indicates standard error (***p < 0.001). **d:** Control or siPCAT-1 treated tumor lysates were subjected to Western blot analysis for c-Myc and AKT1 using specific antibodies. The membrane was reprobed with antibody to actin as an internal control. Right panel shows quantitative representation of Western blot band intensities using Image-J software. Small bar indicates standard error (*, *p* < 0.05; ** *p* < 0.01). **e:** Control or siPCAT-1 treated tumor lysates were subjected to Western blot analysis for PARP using a specific antibody. The membrane was reprobed with antibody to actin as an internal control. **f:** Schematic diagram showing effect PCAT-1 knockdown on c-Myc -AKT1- p38 MAPK signalling pathway in regulation of cell proliferation and apoptosis. Solid arrows indicate activation and blunt arrows indicate inhibition. Dotted arrows indicate upregulation and downregulation following inhibition of PCAT-1 by siRNA

Since we observed the down-regulation of c-Myc and AKT1 and induction of apoptosis following knockdown of PCAT-1 in HNSCC cells, we examined the therapeutic efficacy of PCAT-1. For this, we implanted JHU029 cells into flanks of nude mice. When the tumors were palpable, we randomly divided the mice in two groups. Control or siRNA to PCAT-1 (siPCAT-1) was injected every 4 days for 4 weeks into the tumors**.** Tumor volume was measured weekly. We observed that targeted inhibition of PCAT-1 reduces tumor growth in the mice (Fig. 5a & b). Fluid formation was also observed specially in the control mice which we noted previously [19], and fluid from the outgrowth came out creating tumor measurement sometimes difficult. Mice were sacrificed at 8 weeks of post HNSCC cell implantation, and tumors were extracted. PCAT-1 expression was examined in treated group and control group. We observed significant downregulation of the PCAT-1 in tumors from siRNA treated mice as compared to that of control mice (Fig. 5c). A significant downregulation of c-Myc and AKT1 (Fig. 5d), and enhancement of cleaved PARP (Fig. 5e) was observed in siPCAT-1 treated tumors as compared to control tumors. Thus, our results demonstrated that targeting PCAT-1 inhibits HNSCC tumorigenesis and induces apoptosis.

## Discussion

Our study demonstrated that depletion of PCAT-1 in HNSCC cell lines inhibits cell proliferation and induces apoptosis by i) inhibiting c-Myc and AKT1 expression, ii) activating ASK1 mediated p38 MAPK signalling, and iii) inducing Caspase 9 and PARP cleavage. We also observed that introduction of siRNA to PCAT-1 reduces HNSCC tumor growth in preclinical model.

PCAT-1 was initially identified as an oncogene in prostate cancer [5, 6]. Higher expression of PCAT-1 has been observed in several cancers including colorectal, gastric, esophageal, osteosarcoma, lung, bladder, cervical, hepatocellular carcinoma, cholangiocarcinoma, and multiple myeloma [7]. We observed up-regulation of PCAT-1 in HNSCC patient samples compared to adjacent non-tumor tissues. c-Myc is ubiquitous transcription factor essential for cell proliferation, survival, differentiation, and metabolism in cancers [20]. The AKT pathway is one of the most commonly dysregulated pathways in several cancers [21]. We observed that c-Myc and AKT1 positively correlate with PCAT-1 expression in HNSCC.

LncRNA PVT1, closely located in the c-Myc gene, also regulates c-Myc expression and knockdown of PVT1 resulted in inhibition of cell proliferation and induction of apoptosis in hematologic malignancy via down-regulation of c-Myc [22]. Although Myc inhibition is a powerful approach for cancer therapy, due to its ‘undruggable’ protein structure, targeted therapy has been facing challenges for decades [20]. Therefore, it is plausible that targeting PCAT-1 might have potential for Myc associated cancer therapy. Myc function is dependent on Myc/ MAX complex formation and tumor suppressor protein Mad is recognized as an important cellular antagonist of Myc [23]. Activation of AKT- MAPK signalling was found to induce Mad degradation by phosphorylation resulting functional activation of Myc [23]. Thus, the down-regulation of c-Myc expression may be due to down regulation of AKT1 gene and this mechanism needs to be elucidated in future studies. Modulation of AKT1 gene was noted in the transcriptomic data of PCAT-1 depleted prostate cancer cells [6]. Activation of AKT signalling is a frequent event in human cancers including HNSCC. AKT kinases regulate several important oncogenic events such as cell proliferation, survival, metastasis and angiogenesis [24–26]. Proliferation and survival signal in cancer is mainly achieved by AKT-MAPK signalling which is also responsible for the drug resistant phenotype [24, 25]. Thus, targeting AKT signalling has potential benefit in cancer therapy. AKT1 inactivates ASK1 by phosphorylating at Ser83 residue. Inhibition of AKT enhances p38 MAPK activation through phosphorylation by ASK1 [10–14]. Activation of p38 MAPK is also correlated with apoptosis in prostate or colon cancer [15, 27]. AKT1 inhibition by PCAT-1 depletion resulted in p38 MAPK activation via ASK1, resulting in Caspase 9 mediated HNSCC cell apoptosis.

## Conclusions

Our results for the first time demonstrated an important role of lncRNA PCAT-1 in HNSCC proliferation and apoptosis in in-vitro and in-vivo systems. Depletion of PCAT-1 reduced c-Myc and AKT1 expression resulting in activation of p38 MAPK signalling and induction of apoptosis (Fig. 5f). Thus, targeting PCAT-1 will have therapeutic advantages against HNSCC.

## Abbreviations

ASK1: Apoptosis signal- regulating kinase 1
GAPDH: Glyceraldehyde-3-phosphate dehydrogenase
HNSCC: Head and neck squamous cell carcinoma
lncRNA: Long non-coding RNA
Mad: MAX dimerization protein 1
MAPK: Mitogen-activated protein kinase
MAX: MYC Associated Factor X
PARP: Poly (ADP-ribose) polymerases
PCAT-1: Prostate cancer associated transcript 1
PVT1: Plasmacytoma variant translocation 1
